# Detection of Retroviral Super-Infection from Non-Invasive Samples

**DOI:** 10.1371/journal.pone.0036570

**Published:** 2012-05-08

**Authors:** Adeelia S. Goffe, Anja Blasse, Roger Mundry, Fabian H. Leendertz, Sébastien Calvignac-Spencer

**Affiliations:** 1 Research Group Emerging Zoonoses, Robert Koch-Institut, Berlin, Germany; 2 Wildlife Conservation Research Unit, University of Oxford, Oxford, United Kingdom; 3 Department of Primatology, Max Planck Institute for Evolutionary Anthropology, Leipzig, Germany; Institut Pasteur Korea, Republic of Korea

## Abstract

While much attention has been focused on the molecular epidemiology of retroviruses in wild primate populations, the correlated question of the frequency and nature of super-infection events, *i.e.,* the simultaneous infection of the same individual host with several strains of the same virus, has remained largely neglected. In particular, methods possibly allowing the investigation of super-infection from samples collected non-invasively (such as faeces) have never been properly compared. Here, we fill in this gap by assessing the costs and benefits of end-point dilution PCR (EPD-PCR) and multiple bulk-PCR cloning, as applied to a case study focusing on simian foamy virus super-infection in wild chimpanzees (*Pan troglodytes*). We show that, although considered to be the gold standard, EPD-PCR can lead to massive consumption of biological material when only low copy numbers of the target are expected. This constitutes a serious drawback in a field in which rarity of biological material is a fundamental constraint. In addition, we demonstrate that EPD-PCR results (single/multiple infection; founder strains) can be well predicted from multiple bulk-PCR clone experiments, by applying simple statistical and network analyses to sequence alignments. We therefore recommend the implementation of the latter method when the focus is put on retroviral super-infection and only low retroviral loads are encountered.

## Introduction

The genetic diversity of African primate retroviruses has attracted considerable research efforts, some of which notably led to the identification of the original reservoir of the human immunodeficiency viruses in west and central African primates [1]. Many of these works have also allowed the rough determination of prevalence rates for a number of retrovirus/host systems [2]. Simian immunodeficiency viruses (SIV), simian T-cell leukaemia viruses type 1 (STLV-1) and simian foamy viruses (SFV) could all be shown to reach high prevalence rates (>50%) in at least some of their host species [2]–[6]. In these conditions, it seems reasonable to predict that retroviral super-infection, here defined as the simultaneous infection of the same individual host with several strains of the same virus, will occur frequently in (at least) some wild primate hosts.

Retroviral super-infection could significantly contribute to shaping in-host retroviral diversity, which otherwise mainly depends on in-host accumulation of mutations, *i.e.*, the development of quasispecies. Even rare super-infection events might have a tremendous biological significance. Those may indeed bring together retroviral strains far more divergent than those found within quasispecies and result in major recombination-based genome shuffling, the very process that for example gave rise to the simian precursor of HIV-1 [7]. Despite its theoretical importance, the occurrence of retroviral super-infection in wild primate hosts essentially remains an untested hypothesis.

While investigating co-infection with retroviruses belonging to distinct genera does not raise any specific technical difficulty since independent, genus-specific serological/molecular methods will often be used (*e.g.*, [2], [8]), studying super-infection appears more challenging. Indeed, even though direct sequencing or heteroduplex mobility assays of bulk-PCR products might allow detecting heterogeneous retroviral populations of a same species, *e.g.*, HIV-1 subtypes or variants [9], [10], a truly complete depiction of bulk-PCR product contents will require using some kind of dissection method. For the latter purpose, cloning and sequencing or next generation sequencing (NGS) can be implemented. Both methods are, however, known to be prone to *Taq*-induced errors while cloning can result in selective biases since some sequences will be more likely to be cloned than others [11]. This recently prompted the rise of end point dilution PCR (EPD–PCR) - which is independent of preliminary bulk-PCR amplification - as the gold standard for the depiction of intra-individual retroviral diversity [11], [12]. Within this framework, EPD-PCR was successfully applied to the investigation of HIV-1 micro-evolutionary trends (*e.g.*, [13]), including the detection of super-infection events (*e.g.*, [14]).

EPD-PCR might therefore be considered a promising tool for the detection of super-infection cases among wild primate hosts. There is however at least one predictable barrier to its implementation in this context. As retrovirus-oriented studies of wild primate populations essentially rely on the analysis of non-invasively collected samples (most often faeces, *e.g.*, [4], [15]), it is conceivable that samples will only contain retroviral/proviral genomes in minute amounts [16]. Acquiring a substantial sample of EPD-PCR sequences might therefore require prohibitively large amounts of, by definition, rare starting material [17]. Thus, applying initially biased methods might sometimes be preferable as long as the tools exist to extract biological signal from biased datasets.

In the present study, we investigate super-infection with simian foamy viruses (SFV) in wild western and eastern chimpanzees (*Pan troglodytes verus* and *P. t. schweinfurthii*). We first show that, while SFV infection occurs in these ape populations, SFV retroviral/proviral loads, as quantified from faeces, are extremely low. Making use of this example, we then investigate in greater detail the costs and benefits associated to EDP-PCR and concurrent bulk-PCR based methods when only low quality samples are available whereby the explicit focus is placed on the detection of super-infection.

## Materials and Methods

### Sample Description

Faecal samples were collected from two habituated communities of chimpanzees living in western and eastern Africa - *P. t. verus* from Taï National Park, Côte d’Ivoire and *P. t. schweinfurthii* from Budongo Forest Reserve, Uganda. All necessary permissions were obtained for the described field studies - from the Ministry of the Environment and Forests, the Ministry of Research and the directorship of the Taï National Park for the study site in Côte d’Ivoire and from the Uganda Wildlife Authority and the Uganda National Council for Science and Technology for the study site in Uganda. Faecal samples were placed on ice (Taï samples) or soaked in RNAlater (Qiagen, Hilden, Germany; Budongo samples) directly after collection, before being stored in liquid nitrogen (Taï samples) or at −20°C (Budongo samples), as previously described [17], [18]. Because primary infection with SFV is assumed to occur in early adulthood [4], [19], samples were selected from individuals older than ten years of age at the date of collection (range: 15–42 years). All details about individual samples are given in Table S1.

### Molecular Biology Analyses

#### Nucleic acids’ extraction and cDNA generation

DNA and RNA were co-extracted with the GeneMATRIX Stool DNA Purification Kit (Roboklon GmbH, Berlin, Germany) using 80 mg faeces (Taï samples) or 100 µL homogenate (Budongo samples) and 5 µl carrier RNA (Qiagen, Hilden, Germany) to enhance RNA yield. First-strand cDNA synthesis was then performed using SuperScript™ II RT (Invitrogen™, Karlsruhe, Germany) with random hexamer primers. We did not consider RNA genomes as our specific targets. Therefore, extracts were not treated with DNAse so as to allow for the detection of SFV DNA (either packaged in viral particles or proviral), would it occur in faecal samples. Though published results suggest that SFV DNA will be shed much less frequently in faeces than SFV RNA [4], it should be considered that any sequence produced here might come from RNA or DNA genomes found in viral particles or shed infected cells or from proviral DNA.

#### Initial PCR screening

To identify positive faeces, a nested PCR assay using a set of generic primers targeting a 470 base pair (bp) fragment of the integrase (int) gene was employed [first round primers: PFVint1s, 5′-GCCACCCAAGGGAGTTATGTGG-3′, PFVint2as, 5′-GCTGCACCCTGATCAGAGTG-3′; second round primers: PFVint3s, 5′-CCTGGATGCAGAGTTGGATC-3′, PFVint4as, 5′-GAAGGAGCCTTAGTGGGGTA-3′; 4,20], using 1.5 µl cDNA as template in a 15 µl reaction mixture containing Platinum® Taq DNA-Polymerase (Invitrogen™, Karlsruhe, Germany). Among many positive samples, six (obtained from two males and four females from TNP) were then tested with a TaqMan probe-based quantitative assay targeting another short fragment of the polymerase gene (pol; primers sense, 5′-CTTCAACCTTTGCTGAATG-3′, and antisense, 5′-TAATACAGGGCTATAGGTGT-3′; TaqMan probe 6′-FAM-TTGGAATTCAGTACTCCTTATCACCC-3′-BHQ1), using 2 µl cDNA as template in reactions otherwise prepared as in [21]. This assay was also tested on dilutions of plasmids containing the corresponding sequence and could detect <5 molecules per reaction, including when plasmids were mixed in “faecal” SFV-negative cDNA (data not shown). The final sample set examined in this study included these six samples as well as four positive faeces samples from Budongo (obtained from two males and two females). The latter were not tested with qPCR as they were only available as RNAlater homogenate, which prevented determining the mass of faecal matter effectively used (faecal matter mass was not determined at the time of collection).

#### End-point dilution PCR

Several dilutions of cDNA were tested for each isolate so as to identify the dilutions resulting in success rates <30%, conditions in which about 80% of the sequences can be predicted to stem from a single starting template molecule [11]. For each individual, 15 EPD-PCR products were sequenced on both strands according to the Sanger’s method. A fasta file comprising all unique EPD-PCR sequences is available at http://www.sebastiencalvignac.fr.

#### Bulk-PCR and cloning

Five positive bulk-PCR products were also obtained from each individual, using the same aforementioned nested PCR assay, which was run using 1.5 µL undiluted cDNA as template (cDNA was always derived from the same faecal sample as used for EPD-PCR). All resulting PCR products were sub-cloned using a Topo TA cloning kit (Invitrogen™, Karlsruhe, Germany) according to the manufacturer’s instructions. Following colony PCR and visualisation on a 1.5% agarose gel, five positive colony PCR products from each of the amplicons were purified and sequenced on both strands according to the Sanger’s method, thereby generating 25 clone sequences per individual. It should be noted here that we purportedly chose to target a conserved region of the SFV genome and to use a normal (as opposed to high-fidelity) *Taq* polymerase to produce the amplicons to be analysed. Our rationale for that was that, by maximizing *Taq*-induced noise in a context of limited diversity, we would provide the harshest possible substrate for the intended statistical and network analyses.

### Sequence Analysis

#### Sequence processing and preliminary analyses

Chromatograms were analysed using SeqMan®II (DNASTAR® Lasergene™ DNAStar Inc., USA). Sequences comprising mixed bases were discarded. The 400 unique sequences that constituted the final dataset were aligned by eye using SeaView v4 [22], before being checked for evidence of recombination using the RDP, GENECONV, MaxChi, Chimaera, SiScan, and 3Seq tools, as available in RDP3 v3.42 [23]. No recombination event could be detected using these methods (but it should be kept in mind that the short length and limited genetic diversity of the sequences generated here limits the power of many of these tests).

For the 10 individual alignments, observed distances were first calculated with SeaView. The phylogenetic signal content of each alignment was then inspected using likelihood mapping, as implemented in TREE-PUZZLE [24]. Phylogenetic analyses were also performed in a maximum-likelihood framework, using PhyML v3.0 [25] as implemented in SeaView and approximate likelihood ratio test values as branch support values [26]. For all datasets and analyses, a same model of nucleotide substitution was employed (global time reversible plus rate heterogeneity; GTR+G).

To specify the origin of the SFV sequences phylogenetic analyses were also conducted on large datasets including all sequences obtained from a given sup-species (*P. t. verus* or *P. t. schweinfurthii*) to which representative sequences from SFV infecting other great apes were also added. These control analyses were run under maximum likelihood and Bayesian frameworks, using the model of nucleotide substitution pointed at by the best Akaike’s information criterion score (*i.e.*, GTR+G), as determined by jModelTest v.0.0.1 [27]. PhyML was run on a webserver [28], using bootstrap pseudo-replicates (n = 500) as a way to investigate branch robustness. Bayesian analyses were performed using BEAST v1.6.1 [29]. Output of BEAST analyses were examined with companion softwares (available at http://beast.bio.ed.ac.uk/Main_Page), which notably allowed for the computation of branch posterior probabilities, which were taken as branch robustness measures. Both maximum likelihood and Bayesian analyses supported the expected scheme of sub-species specific distribution of SFV [4] (data not shown).

### Statistical Analyses

Our first objective was to develop a statistical tool that provides an objective criterion for classifying individuals as single or super-infected, based on the analysis of a multiple bulk-PCR product clone alignment. We chose to use the shape of the frequency distribution of the number of mismatches derived from an individual clone alignment (thereafter termed ‘mismatch distribution’) as a way to investigate the nature of the underlying infection process. Briefly, our assumption was that in case of single infection, mismatch distribution should be approximately unimodal and can be fit with “unimodal” distribution laws. On the contrary, we expected that super-infection would result in bi- or multimodal distributions and should therefore better fit “bimodal” distribution laws.

We first produced mismatch distributions for all individual EPD-PCR sequence alignments and identified the individuals as cases of single or super-infection. At that stage, assignment was made by eye only as the limited number of variants precluded any statistical assessment of the shape of the distributions.

We then investigated the shape of the much more variable mismatch distributions derived from bulk-PCR product clone alignments. To objectively measure whether (or to what extent) such a distribution was bimodal, we once assumed that the distribution was from a single (normal or Poisson) distribution and once assumed that it was from two normal distributions with equal standard deviations but different means or from two Poisson distributions. For all four assumptions we determined the most likely mean or means and the most likely standard deviation (in case of the normal distribution(s) assumption) using maximum likelihood. We then calculated Akaike’s information criterion, corrected for small samples (AICc) as a measure of model performance [30] and selected the model with the smallest AICc as the most likely mismatch distribution (uni- or bi-modal normal or Poisson distribution). However, when the smallest AICc-value differed by no more than two from the second smallest, we chose the simplest model (*i.e.*, the one with the smallest number of estimated parameters) from all models with AICc-values differing from the smallest AICc-value by at most two (in case the model with one normal distribution and the model with two Poisson distributions both fulfilled this criterion, we chose the one with the smaller AICc). For each individual, we applied this approach to any possible combination of bulk-PCR product clone alignments; *i.e.,* to the only possible combination of all five bulk-PCR product clone alignments (ABCDE), to the five possible combinations of four bulk-PCR product clone alignments (ABCD, ABCE, ABDE, ACDE and BCDE), and so on. In a few cases only two different numbers of mismatches were found in a sample. In these cases we assessed uni−/bimodality based on visual inspection of the distribution because it was impossible to fit the two means model to such a distribution.

We also applied this approach to simulated cases of triple infection. The latter consisted of robust clone “clouds” (supported by both phylogenetic and network analyses, from Taï specimens only) which were shuffled randomly so as to form 84 “triple infection clone alignments”.

All analyses were conducted in R (version 2.10.1, R Development Core Team 2010) using scripts written by one of us (RM).

### Network Analyses

Sequences generated with EPD-PCR are in principle *Taq*-error free sequences [11]. This is not the case of clone sequences, which will on the contrary often harbour singleton changes due to *Taq* errors. We were therefore also interested in defining a method that would allow for the identification of biological sequences potentially comprised in clone alignments (including minor variants) and more particularly of those sequences which might be at the origin of the infection and/or have become main components of the overall retroviral population (hereafter called founder sequences).

Founder sequences with respect to their biological variants-– but also biological sequences with respect to their *Taq*-modified variants – are expected to exhibit two distinctive properties: i) high connectivity and ii) high frequency. While phylogenetic analyses cannot capture these characteristics, tokogenetic analyses (*i.e.* network building) can in principle allow for their simultaneous assessment [31]. TCS [32], which implements statistical parsimony-based network building as initially described in an article by Templeton, Crandall and Sing (1992; [33]), offers the advantage to produce “outgroup probabilities” (OP), a statistic which summarizes sequence connectivity and frequency for all sequences examined.

We first identified EPD-PCR sequences having the highest OPs (either the very best if single infection or the two best ones if super-infection) and checked whether highest OPs derived from clone alignments pointed at the same founder sequences. We also checked whether the appearance of a sequence in several of the five PCR products (ability to replicate) was likely to point at it being a founder sequence. We determined OP values from all possible alignments derived from 3 or 4 PCR products only and in which no sequence was appearing in more than one PCR product so as to determine whether OPs were pointing at EPD-PCR founder sequences when ability to replicate could not be used as a criterion.

Finally, we also built TCS networks for 9 of the 84 simulated triple infection cases and checked whether the three highest OPs were pointing to the appropriate “clouds” of sequences.

For all alignments, median-joining networks [34] were also reconstructed using Network v4.610 (www.fluxus-engineering.com). Those were always similar in shape to their statistical parsimony counterparts. Median-joining networks are presented in this article, as their layout could be much more easily reworked, using a dedicated tool, Network Publisher (www.fluxus-engineering.com).

## Results

### Quantification of SFV Retroviral Loads in Faeces

Quantitative PCR performed on samples identified as positive by a nested PCR approach resulted in negative results for the six samples tested, even though the former test exhibited a high sensitivity (detection threshold <5 molecules). When extrapolating to the 80 mg faeces typically used for extraction, this suggested that SFV retroviral/proviral loads in faeces are in the range of at maximum a handful of copies per mg faeces.

### EPD-PCR

For each selected sample, we aimed at acquiring a minimum of 15 EPD-PCR sequences, a point at which the average nucleotide distance most often reached a plateau, thereby suggesting appropriate sampling of the underlying viral population (Figure S1). Moderate dilutions (down to a maximum factor of 20) were always sufficient to lower the success rate to less than 30% of the reactions (Table S1) [11]. On average, 69.3 µL of undiluted cDNA was necessary to gather the desired number of EPD-PCR sequences, though the range of used material was highly variable (median volume: 47 µL, range: 11–179 µL; Table S2).

**Figure 1:**
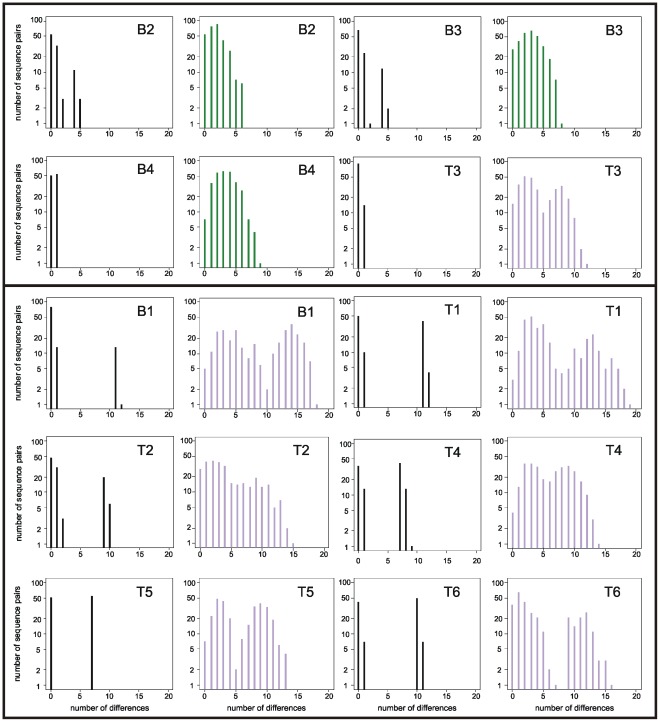
Mismatch distribution analyses of EPD-PCR and clone sequence datasets. Individuals are ordered such that first four individuals with a single infection (as determined by EPD-PCR) are shown (B2, B3, B4 and T3; above the bar) followed by individuals with super-infections (below the bar). Plots are paired for each individual with the EPD-PCR dataset on the left and the bulk-PCR clone dataset on the right. EPD-PCR distributions are black; bulk-PCR clone distributions identified as unimodal (ΔAICc<2) are green; bulk-PCR clone distributions identified as bimodal are purple (ΔAICc>2).

In all cases, several unique SFV sequences were identified by EPD-PCR (range: 2–5). These sequences were all assumed to represent authentic biological variants and therefore directly used to make a decision regarding chimpanzee-specific SFV super-infection, using mismatch distributions. This way, eight of the 10 individuals could be unambiguously assigned as single or super-infected (6/6 *P. t. verus*: T1 to T6, 2/4 *P. t. schweinfurthii*: B1 and B4; Figure 1). The two remaining individuals (B2 and B3) exhibited slightly ambiguous distributions but were ultimately assigned to the single infection category, based on a rather small maximum divergence of 5 bp (as compared to a minimum of 7 bp difference between different strains and a mean of 10 bp for unambiguously super-infected individuals).

### Bulk-PCR Product Cloning and Sequencing

For each sample we generated five bulk-PCR products, consuming a median volume of 12.5 µL undiluted cDNA. All resulting PCR products (named A to E) were sub-cloned and five clones were sequenced per product. Clone alignments typically exhibited higher levels of polymorphism than the corresponding EPD-PCR sequence alignments, an expected consequence of the accumulation of random *Taq*-errors (*e.g.*, Figure S2).

Simple statistics computed from individual alignments, such as the mean observed distance among clones or the percentage of resolved/unresolved topologies obtained using quartet puzzling analysis (*i.e.*, likelihood mapping), did not correlate well with infection status as determined through EPD-PCR sequence analysis, even though trends could be observed (Table S3). In addition, the proportion of unresolved quartets in the likelihood mapping analyses (32.5–98.2%; Table S3) suggested alignments only comprised little phylogenetic information.

**Figure 2:**
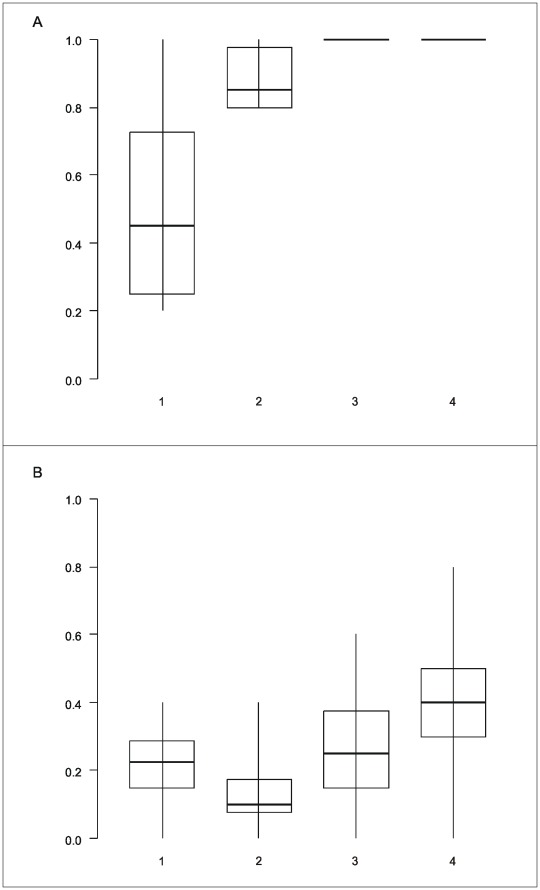
False negativity and positivity as a function of the number of bulk-PCR products analysed. (a) Probability to detect bimodality (*i.e.*, super-infection) in the frequency distribution of the number of mismatches per pair of sequences as a function of the number of bulk-PCR products analysed and for subjects for which EPD-PCR revealed a super-infection (n = 6); (b) Probability to detect bimodality in the frequency distribution of the number of mismatches per pair of sequences as a function of the number of bulk-PCR products analysed and for subjects for which EPD-PCR revealed a single infection (n = 4). Shown are median, quartiles, minimum and maximum, of the respective probabilities per subject.

Assessing the shape of the mismatch distribution (uni- or bi-modal) for the 10 complete clone alignments (*i.e.,* all 25 sequences stemming from five bulk-PCR products of a subject), allowed to properly infer individual status with respect to super-infection, as revealed by EPD-PCR results, in 9 out of ten cases (Figure 1). Using the same test it was also possible to investigate the impact of the number of bulk-PCR products analysed on the super-infection diagnostic. The latter revealed that the probability to infer single infection for super-infected individuals (*i.e.*, probability of a false negative) was zero as soon as three or more products were analysed (Figure 2a). The probability to infer super-infection for single infected individuals (*i.e.*, probability of a false positive) was markedly higher (up to 80%; Figure 2b).

### Identification of Founder Sequences

Sequences having likely founded the sampled retroviral populations (or at least standing for major variants at the time of sampling) were identified from EPD-PCR sequence alignments using outgroup probabilities (OPs) generated by TCS. These EPD-PCR founder sequences were actually captured in 14 of 16 cases (here one case equals one founder sequence), when considering the total five PCR products generated for each sample.

From these 14 clone sequences, 10 had been replicated. Replication was generally not observed for other clone sequences, with two exceptions. In the first case, two distinct clone sequences determined from B4 were replicated (B4 had been identified as single infected). Both sequences had been sampled through EPD-PCR and were very closely related (1 bp divergence). In the second case, a T1 clone sequence could be replicated which had not been identified through EPD-PCR, thereby revealing a possible example of erroneous replication-based identification of EPD-PCR sequences or the sampling of a biological variant not sampled by EPD-PCR.

**Figure 3:**
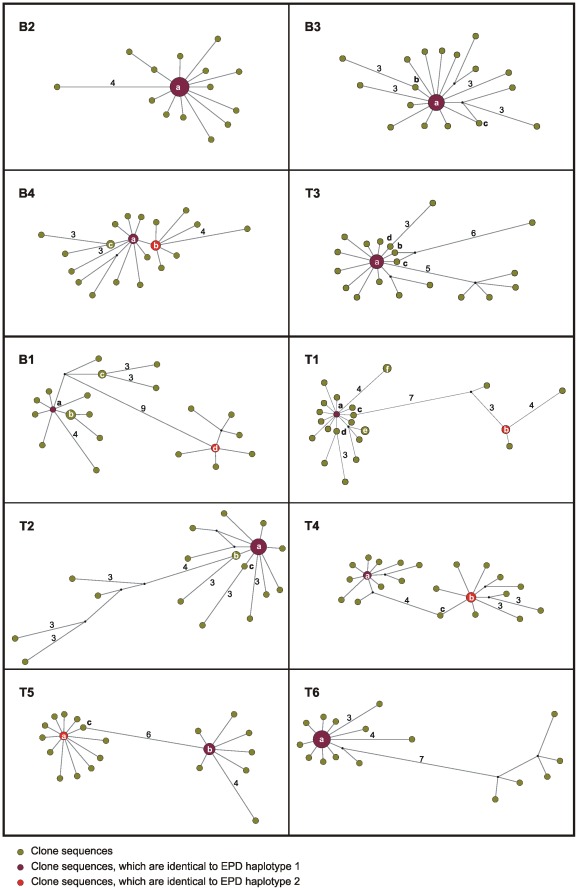
Median joining network of bulk PCR product clone sequences. As in Figure 1, the networks are ordered by infection status. Within each network, node size is proportional to the frequency of sequence occurrence (total n = 25 for each individual). Branch lengths are directly related to the number of mutations between sequences, with values noted for differences greater than two base pairs. Clone haplotypes a–f (as shown in Table 1) are noted within or adjacent to their corresponding node. Networks generated using TCS were highly similar (data not shown).

TCS networks were also computed from all clone alignments in order to assess the performance of OPs in pointing at EPD-PCR-determined founder sequences. The overall shape of the clone networks reflected expectations; “star-like” patterns were commonly observed, thereby highlighting founder effects (both biological and *Taq*-error dependent; Figure 3). Super-infected individuals also exhibited more complex networks, which were sometimes even disconnected (when computed using statistical parsimony, data not shown; Figure 3). Phylogenetic analyses of individual datasets always provided high support to the branch defining distinct clouds of sequences in networks of super-infected individuals (Table S3). In all the 14 aforementioned cases where EPD-PCR-determined founder sequences had been sampled, the highest or two highest OP values, for single and super-infected individuals, respectively, highlighted the correct clone sequences as founders (Table 1). The co-occurrence of two very closely related sequences in B4 was also supported by OPs (Table 1). In the two remaining cases - where one of the two EPD-PCR founder sequences had not been captured by cloning – selecting the sequences with the two highest OPs as potential founder sequences involved would have resulted in: i) misleadingly identifying a sequence closely related to the sampled EPD-PCR founder sequence as the source of the second infection (clone b from T2; Figure 3 and Table 1); and ii) being unable to identify a second founder sequence (T6; Figure 3 and Table 1), as all sequences differing from the first founder sequence were only exhibiting the “noise” OP, *i.e.,* the minimal OP value attributed to poorly represented/connected sequences.

**Table 1:** Identification of founder sequences from EPD-PCR and bulk-PCR clone sequence alignments.

Individual	EPD network			Clone network		
	EPD-PCRhaplotypes	Number ofsequences	HaplotypeOP	Bulk-PCRclonehaplotypes	HaplotypeOP	Number and originof sequences^#^
B1*	**1**	**13**	**0,93**	***a***	0,36	**1×B**
	**−**	**−**	**−**	*b*	0,26	3**×**C
	**−**	**−**	**−**	*c*	0,1	2**×**A
	**2**	**1**	**1**	d^*^	0,5	**2×E**
	3	1	0,07	**−**	**−**	**−**
B2	**1**	**11**	**0,88**	***a***	0,48	**2×A, 4×B, 2×C, 1×D, 2×E**
	**−**	**−**	**−**	*b*	0,35	1**×**E
	2	1	0,03	**−**	**−**	**−**
	3	1	0,03	**−**	**−**	**−**
	4	1	0,03	**−**	**−**	**−**
	5	1	0,03	**−**	**−**	**−**
B3	**1**	**12**	**0,9**	***a***	0,33	**3×B, 1×C, 2×D, 2×E**
	**−**	**−**	**−**	*b*	0,23	1**×**C
	**−**	**−**	**−**	*c*	0,23	1**×**D
	2	1	0,03	**−**	**−**	**−**
	3	1	0,03	**−**	**−**	**−**
	4	1	0,03	**−**	**−**	**−**
B4	**1**	**9**	**0,6**	***a***	0,35	**1×B, 1×C, 1×E**
	2	6	0,4	*b*	0,26	2**×**D, 1**×**E
	**−**	**−**	**−**	*c*	0,15	2**×**A
T1*	**1**	**10**	**0,9**	***a***	0,37	**1×E**
	**2**	**4**	**1**	***b***	0,11	**1×A, 1×D**
	**−**	**−**	**−**	*c*	0,07	1**×**E
	**−**	**−**	**−**	*d*	0,07	1**×**C
	**−**	**−**	**−**	*e*	0,04	1**×**B, 1**×**D
	**−**		**−**	*f*	0,04	2**×**A
	3	1	0,09	**−**	**−**	**−**
T2*	**1**	**10**	**0,89**	***a***	0,33	**1×B, 3×D, 4×E**
	**−**	**−**	**−**	*b*	0,26	2**×**B
	**−**	**−**	**−**	*c*	0,23	1**×**A
	2	1	0,03	**−**	**−**	**−**
	3	1	0,03	**−**	**−**	**−**
	4	1	0,03	**−**	**−**	**−**
	**5**	**2**	**1**	**−**	**−**	**−**
T3	**1**	**14**	**0,93**	***a***	0,29	**2×C, 2×D, 2×E**
	**−**	**−**	**−**	*b*	0,17	1**×**B
	**−**	**−**	**−**	*c*	0,17	1**×**B
	**−**	**−**	**−**	*d*	0,17	1**×**E
	2	1	0,07	**−**	**−**	**−**
T4	**1**	**7**	**0,5**	***a***	0,32	**2×A**
	**2**	**6**	**0,44**	***b***	0,19	**2×D, 1×E**
	**−**	**−**	**−**	*c*	0,05	1**×**E
	3	1	0,03	**−**	**−**	**−**
	4	1	0,03	**−**	**−**	**−**
T5	**2**	**6**	**0,4**	***a***	0,33	**1×C, 1×E**
	**1**	**9**	**0,6**	***b***	0,22	**2×B, 2×D**
	**−**	**−**	**−**	*c*	0,11	1**×**E
T6*	**1**	**7**	**0,88**	***a***	0,74	**2×A, 2×B, 1×C, 2×D, 2×E**
	2	1	0,13	**−**	**−**	**−**
	**3**	**7**	**1**	**−**	**−**	**−**

All EPD-PCR haplotypes are presented, while only those haplotypes getting OP values above the minimum value are shown for bulk-PCR clone sequences. Assumed founder sequences are highlighted in bold. # origin of sequences refer to the bulk-PCR products from which they originate (*e.g.*, if haplotype *a* appeared two times in PCR product B and once in PCR product C, then 2**×**B and 1**×**C will appear in this column) * individuals for which statistical parsimony analyses produced two separated networks; here the sum of all OPs will be greater than one as OPs will be calculated independently for each network.

In order to allow further comparison of replication-based and network/OP-based identification methods, we computed networks for all the possible combinations of three or four PCR products for which EPD-PCR founder sequence(s) would not have been replicated (n = 41) (Table S4). Using OPs, EPD-PCR founder sequences were properly identified in 63% of the cases when only 100% accurate identifications were considered (both founder sequences were properly identified in cases of super-infection; Table S2) and in 77% of the cases when partial identifications were considered as well (only one of the founder sequences was properly identified in cases of super-infection; data not shown).

### Simulated Triple Infections

No triple infection case could be evidenced in our experimental set of samples. We therefore simulated 84 triple infection cases which were all recognized as super-infection cases, including six triplets consisting of two interspersed “clouds” plus one more distant one, *i.e.*, actually cases of double infection similar to those identified in our experimental panel. Nine randomly selected simulated triple infections cases were also examined using TCS: the three highest OPs properly pointed to EPD-PCR sequences from the three “clouds” in all cases.

## Discussion

### EPD-PCR

Our results allow addressing the question of the applicability of EPD-PCR when samples only contain minute quantities of the targeted retrovirus/provirus. Here we show that in such cases the amount of biological material required for proper EPD-PCR analysis can largely exceed the requirements of “classical” standard bulk-PCR assays (with a *ca.* 30-fold increase). This is in sharp contrast with applications of EPD-PCR on samples exhibiting high retroviremia, where requirements in undiluted cDNA might often be comparable to those of a single bulk-PCR assay. The magnitude of the increase poses a serious problem when one considers that limitation of biological material is an inherent characteristic of samples obtained from wild and endangered animal populations [17]. In these conditions, biological material use has to be optimised so as to allow investigating the biology and evolution of the broadest possible spectrum of pathogens. As an example, one could cite the recent use of the same collection of faecal samples from wild-living chimpanzees for the investigation of host [35], SIV [15], SFV [4] and *Plasmodium* [36] genetics. The implementation of EPD-PCR for the investigation of super-infection might therefore be unreasonable where “native” endpoint dilution of pathogen DNA/RNA is to be encountered. In such situations, careful alternatives, which would still allow for a fair depiction of the underlying retroviral/proviral populations, should be favoured.

There are two main alternatives to EPD-PCR analysis: bulk-PCR product cloning and sequencing and NGS. Though the latter strategy would be less labour-intensive and possibly even cheaper [10], we considered it as likely to produce highly redundant information where bulk-PCR amplification starts from only a few template molecules. We therefore focused on bulk-PCR product cloning and sequencing, which we considered appropriately scaled to our objectives.

### Bulk-PCR Product Cloning and Sequencing

Here we show that bulk-PCR product cloning is more parsimonious than EPD-PCR, even when gathering several bulk-PCR products per sample. Thus, in our conditions, producing five bulk-PCR products required *ca.* 4-times less material than performing an equivalent EPD-PCR analysis. Interestingly, using a comparable amount of material, EPD-PCR analyses would have resulted in gathering only 4 sequences, in which case three out of the six aforementioned cases of super-infection, namely B1, T1 and T2, would have faced a high probability of going unnoticed: 56%, 29% and 56%, respectively. This clearly demonstrates that re-scaling EPD-PCR so as to spare material would significantly alter the probability to detect super-infection.

However, bulk-PCR product cloning is itself known to yield a biased view on within host retrovirus/provirus populations, at least when a detailed view is needed (*e.g.*, stoechiometries of viral genomes will likely be significantly skewed) [11]. Here, we aimed at establishing a coherent analytical framework likely to identify super-infection cases and the respective underlying main strains from bulk-PCR product cloning results, even where phylogenetic signal is low (Table S3). Our strategy was two-fold.

We first wanted to establish a statistical method which allows for the quick identification of super-infection cases from clone alignments. We show here that using the shape of the mismatch distribution from clone alignments is a worthy approach. Using clones stemming from five independent bulk-PCR products, we reached a satisfying 90% success rate, *i.e.* rate of agreement with EPD-PCR results (T3 was the only case with disagreement), notably identifying the six super-infected individuals. In addition to yielding robust estimates of the expected results of an EPD-PCR experiment, the implementation of such a statistic also has the advantage to allow for a quick assessment of the number of samples needed to detect super-infection. Here, it suggests that using clones derived from fewer PCR products (n≥3) would still have allowed for reaching a high success rate (100%) but at the cost of a rather large false positive rate (up to 60%). This high false positive rate is however entirely dependent on the inclusion of a single individual, T3, since running the same analysis with sequences stemming from the three other individuals results in a 0% false positive rate (when ≥3 PCR products are considered). Interestingly, T3 was identified as single-infected by EPD-PCR analysis, while the global, five bulk-PCR product-based alignment rather supported a super-infection (the only faulty assignation in our sample). As the “second” strain evidenced by bulk-PCR cloning results only appears in one of the PCR products, T3 might well be a case where bulk-PCR cloning actually revealed a minor variant whose presence went undetected by EPD-PCR. Accordingly, we interpret the abovementioned high false positive rate as likely resulting from a sampling artefact. All in all, this case study therefore illustrates well how a careful use of our statistical approach could help in ascertaining the most efficient bulk-PCR based sampling method, *e.g.*, at the very beginning of larger projects.

The second step of our strategy consisted of identifying biological sequences with a particular focus on founder sequences, here understood as sequences having been at the origin of the infection or as sequences which ultimately became main components of the overall retroviral population. One of the most severe drawback of a bulk-PCR based method when compared to EPD-PCR indeed lies in the fact that, contrary to EPD-PCR sequences, many clone sequences are expected to comprise artifactual mutations [11]. The ability to identify biological sequences from *Taq-*modified ones would further support the use of bulk-PCR derived clone alignments. Theoretically, in-host evolution of retroviruses/proviruses is likely to be well captured by network analyses, which should point at such sequences (assuming they have been sampled). We show here that, in addition to providing an excellent visual support for the analysis of retroviral/proviral evolution, networks can also be used to identify truly biological (as opposed to *Taq*-error modified) founder sequences, through the use of OPs (as implemented in TCS [32]). In our study, the highest OPs in clone networks always pointed at founder sequences which were supported by EPD-PCR results, and, where super-infection was assumed, always identified the two founder sequences, when they had been sampled. Importantly, we also show that network/OP-based analysis actually supersedes replication-based identification of founder sequences, notably exhibiting good performance where no replicated sequence is available. This good performance is in striking contrast with the results that would have provided the only possible alternative method, *i.e.*, phylogenetic analyses. Though it is sometimes argued that in phylogenetic trees ancestral sequences should appear as short branches basal to the longer branches of their descendants [31], it is indeed very unlikely that *Taq*-modified sequences will ever be separated from the biological sequence from which they are derived by a branch receiving strong statistical support (individual datasets only comprised weak phylogenetic information; Table S3). Therefore, networks and TCS-calculated OPs provide a unique, and so far under-explored, opportunity to identify founder sequences out of the *Taq*-induced noise.

The results that we obtain on simulated triple infections (no triple infection case could be identified from our experimental dataset) further support the robustness of our two-step algorithm. Mismatch distributions derived from simulations are indeed always a better fit to a bimodal distribution model, suggesting that our statistical tool actually detects deviation from a unimodal distribution model. On condition that it is known that super-infection involves more than two major type of sequences (which will require visual inspection of the networks), TCS-assessed OPs are also very efficient in identifying more than two likely founder sequences.

Nevertheless, it should be kept in mind that the method discussed here, even though all in all robust, remains bulk-PCR-based. As such, it is potentially heavily influenced by PCR- and/or sub-cloning-induced selective biases. The latter could presumably influence the results in both directions, *i.e.* lead to false negatives when sequences of one of the infecting group of strains will be preferentially amplified and/or false positives when sequences of one minor infecting group of strains will be preferentially amplified. Selective biases are however unlikely to result in the ultimate identification of super-infection where individuals are truly single infected, *i.e.* where no minor super-infection occurs. The main problem would therefore lie in accurately determining the frequency of biologically significant super-infection events, such as those resulting in several relatively distant strains becoming quantitatively important players in the overall retroviral population. This could however be corrected by applying the same approach to several genomic markers at one and the same time, since independent primer pairs are unlikely to result in congruent selective biases.

To our knowledge, SFV super-infection of wild primates has only been reported incidentally a couple of times, also in the case of chimpanzees [4]. Our results therefore provide a first glimpse into the frequency of SFV super-infection in a natural primate/retrovirus system. Even though the size of our host sampling clearly precludes drawing any definitive conclusions, it seems plausible that super-infection of wild chimpanzees with their species-specific SFV is common. Such a high preliminary estimate is both an expected result, since SFV reach very high prevalence rates in the species [4], and a puzzling finding as SFV, like other retroviruses, have evolved mechanisms aimed at restricting super-infection [37]. It clearly underlines how little we know about retroviral super-infection in the wild, and consequently, the necessity for further studies to address this question.

### Conclusions

Here we present an overall methodology that could stand for an acceptable alternative to EPD-PCR analysis for studies which involve the detection of retroviral super-infections from non-invasive samples. SFV has the slowest evolutionary rates of all retroviruses [38], which should result in making the task of identifying super-infection harder than for other members of this viral family. The fact that our method is efficient in unveiling SFV super-infection instances thus offers a good perspective for its implementation for studies focused on other primate retroviruses such as STLV-1 and SIV.

## Supporting Information

Figure S1 **Average number of mismatches between EPD-PCR sequences as a function of the number of sequences considered.** All possible assemblages of two, three, and so on up to 15 sequences were considered. For each of those the mean pairwise distance was computed. The average of all mean pairwise distances was finally plotted. Appropriate sampling of the underlying sequence population can be expected to result in reaching a plateau phase.
(TIF)

Figure S2 **Alignment of EPD-PCR and bulk PCR clone sequences for individual B1.**
(JPG)

Table S1 **Individual sample characteristics.** Samples have been ordered according to the age of the chimpanzee at the time of collection. Individuals whose name starts with a T are *P. t. verus* from Taï National Park, Côte d’Ivoire, with a B *P. t. schweinfurthii* from Budongo Forest Reserve, Uganda. * According to EPD-PCR.
(DOC)

Table S2 **Quantities of material used in the generation of EPD-PCR sequences.** The total numbers of necessary experiments are reported in the left section, and those having finally be performed in EPD-PCR conditions are reported in the right section. * marks individuals for which it was necessary to re-extract from the same original faecal bolus to obtain a sufficient number of EPD-PCR sequences.
(DOC)

Table S3 **Additional statistics computed from individual bulk-PCR clone sequence datasets.** EPD-PCR single infected individuals are greyed, others are super-infected. ^$^ Category “Partly resolved” is not shown here but was always under 2.5% and can be deduced from the other values, “Partly resolved” = 100%-(“Unresolved”+”Resolved”).^ #^ Support is given as approximate likelihood ratio test (aLRT) values and is about the main bipartition observed in corresponding networks. NA: not assessed.
(DOC)

Table S4 **Identification of EDP-PCR founder sequences from bulk-PCR clone sequence analyses where replication-based identification is not applicable.** Only the most conservative view (that showing 100% accuracy in identification) is presented here. Outgroup probabilities (OPs) were computed with TCS for all possible combinations of bulk-PCR clone alignments which would not have allowed using replication as a criterion for EPD-PCR founder sequence identification. * marks a case where the founder sequences built separate networks consisting of less than 3 sequences; these were assumed to be negative.
(DOC)
