# Supplementary figures and images for: Detection of Retroviral Super-Infection from Non-Invasive Samples
Source: PLoS One. 2012 May 8;7(5):e36570. doi: 10.1371/journal.pone.0036570 (PMC3348140; doi:10.1371/journal.pone.0036570)

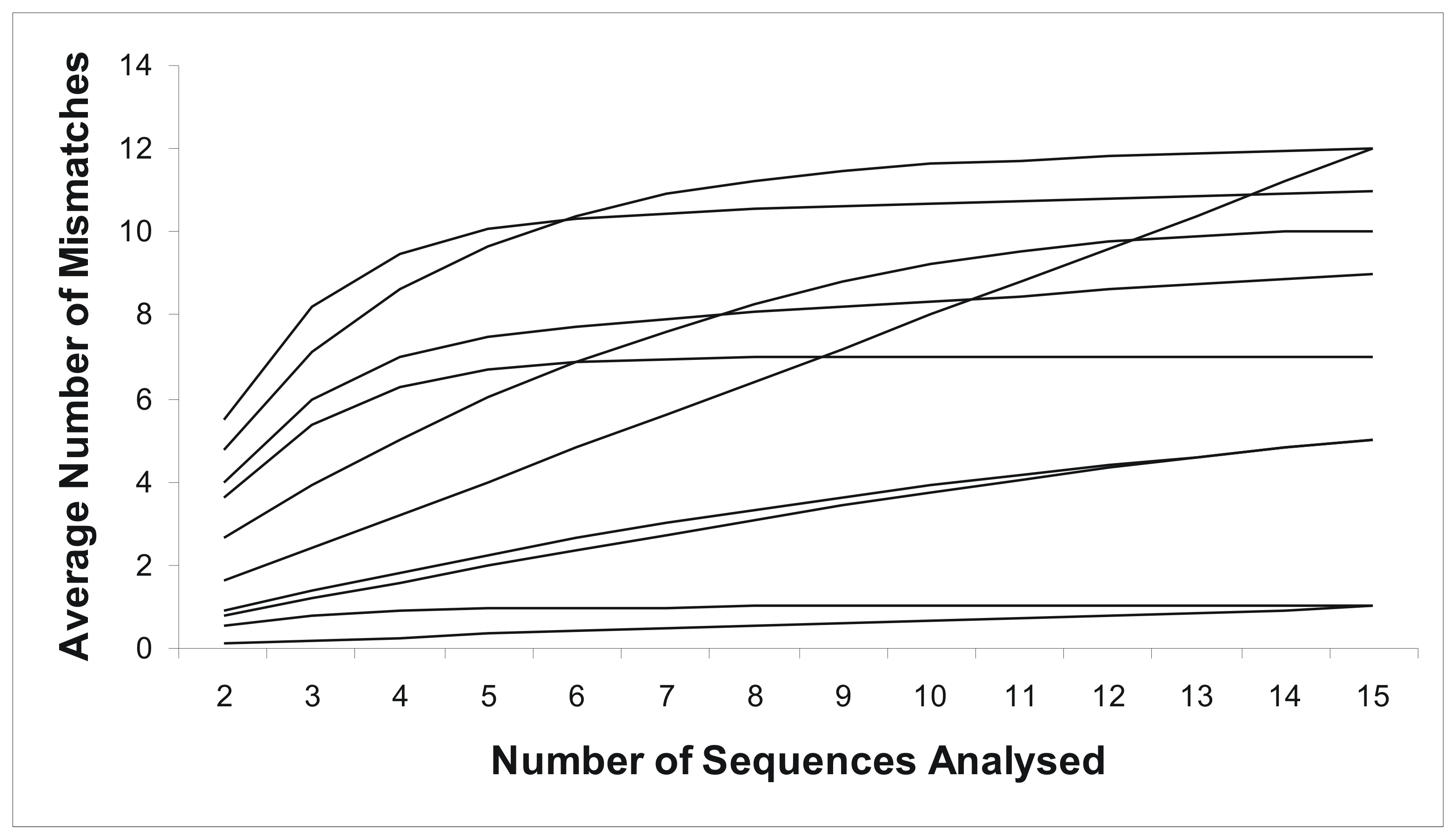

Supplement: Figure S1 — Average number of mismatches between EPD-PCR sequences as a function of the number of sequences considered. All possible assemblages of two, three, and so on up to 15 sequences were considered. For each of those the mean pairwise distance was computed. The average of all mean pairwise distances was finally plotted. Appropriate sampling of the underlying sequence population can be expected to result in reaching a plateau phase. (TIF) [file pone.0036570.s001.tif]

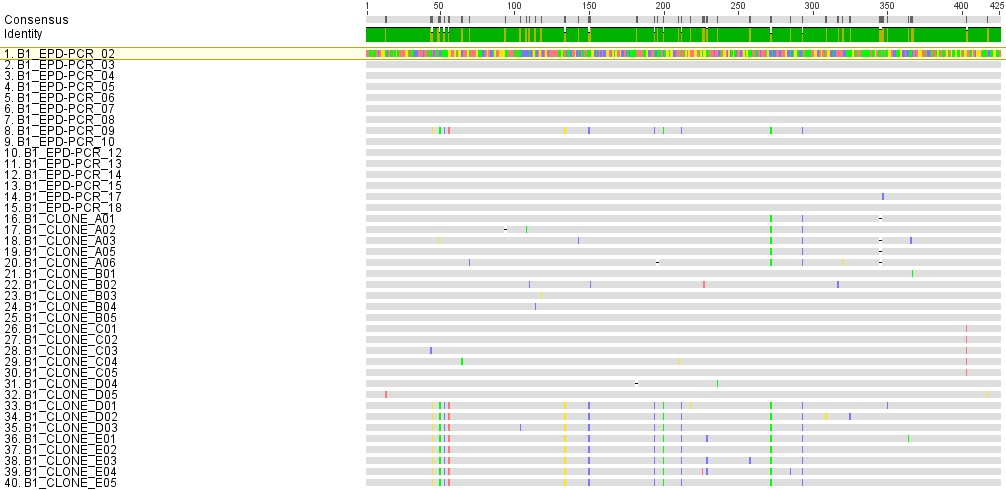

Supplement: Figure S2 — Alignment of EPD-PCR and bulk PCR clone sequences for individual B1. (JPG) [file pone.0036570.s002.jpg]
