# Supplementary material for: Detection of Retroviral Super-Infection from Non-Invasive Samples
Source: PLoS One. 2012 May 8;7(5):e36570. doi: 10.1371/journal.pone.0036570 (PMC3348140; doi:10.1371/journal.pone.0036570)
Supplement: Table S1 — Individual sample characteristics. Samples have been ordered according to the age of the chimpanzee at the time of collection. Individuals whose name starts with a T are P. t. verus from Taï National Park, Côte d’Ivoire, with a B P. t. schweinfurthii from Budongo Forest Reserve, Uganda. * According to EPD-PCR. (DOC) [file pone.0036570.s003.doc]

**Table S1. Individual sample characteristics.**

Samples have been ordered according to the age of the chimpanzee at the time of collection. Individuals whose name starts with a T are *P. t. verus* from Taï National Park, Côte d’Ivoire, with a B *P. t. schweinfurthii* from Budongo Forest Reserve, Uganda. * According to EPD-PCR.

| **Individual** | **Sex** | **Age at sampling** | **Sampling date** | **Birth date** | **Infection status*** |
| --- | --- | --- | --- | --- | --- |
| T3 | male | 17 | 2006 | 1989 | single |
| B1 | male | 17 | 2007 | 1990 | super |
| B2 | female | 17 | 2007 | 1990 | single |
| B3 | female | 24 | 2007 | 1983 | single |
| T1 | female | 25 | 2002 | 1977 | super |
| B4 | male | 26 | 2008 | 1982 | single |
| T5 | female | 34 | 2004 | 1970 | super |
| T2 | female | 35 | 2005 | 1970 | super |
| T4 | female | 40 | 2005 | 1965 | super |
| T6 | male | 42 | 2006 | 1964 | super |
