# Supplementary material for: Detection of Retroviral Super-Infection from Non-Invasive Samples
Source: PLoS One. 2012 May 8;7(5):e36570. doi: 10.1371/journal.pone.0036570 (PMC3348140; doi:10.1371/journal.pone.0036570)
Supplement: Table S2 — Quantities of material used in the generation of EPD-PCR sequences. The total numbers of necessary experiments are reported in the left section, and those having finally be performed in EPD-PCR conditions are reported in the right section. * marks individuals for which it was necessary to re-extract from the same original faecal bolus to obtain a sufficient number of EPD-PCR sequences. (DOC) [file pone.0036570.s004.doc]

**Table S2. Quantities of material used in the generation of EPD-PCR sequences.**

The total numbers of necessary experiments are reported in the left section, and those having finally be performed in EPD-PCR conditions are reported in the right section. * marks individuals for which it was necessary to re-extract from the same original faecal bolus to obtain a sufficient number of EPD-PCR sequences.

|  | Total | | | Dilutions and PCRs within EPD range | | |
| --- | --- | --- | --- | --- | --- | --- |
| Individual | Min / max of dilution | Number of PCRs performed | cDNA (µl) | Min / max of endpoint dilution | Number of PCRs performed | Number of Positive PCR (%)† |
| B1* | original / 1:20 | 168 | 71 | 1:3 / 1:20 | 104 | 21 (20) |
| B2* | original / 1:5 | 218 | 179 | original / 1:5 | 191 | 17 (9) |
| B3 | 1:5 / 1:10 | 112 | 31 | 1:5 / 1:10 | 112 | 15 (13) |
| B4 | 1:3 / 1:5 | 146 | 56 | 1:4 / 1:5 | 130 | 17 (13) |
| T1 | 1:5 / 1:10 | 92 | 16 | 1:10 | 76 | 20 (26) |
| T2 | 1:4 / 1:5 | 93 | 34 | 1:4 / 1:5 | 93 | 22 (24) |
| T3* | original / 1:10 | 298 | 175 | original / 1:10 | 266 | 18 (7) |
| T4* | original / 1:5 | 176 | 82 | 1:5 | 152 | 17 (11) |
| T5 | 1:3 | 76 | 38 | 1:3 | 76 | 15 (25) |
| T6 | 1:5 / 1:20 | 92 | 11 | 1:20 | 76 | 18 (24) |
