# Supplementary material for: Detection of Retroviral Super-Infection from Non-Invasive Samples
Source: PLoS One. 2012 May 8;7(5):e36570. doi: 10.1371/journal.pone.0036570 (PMC3348140; doi:10.1371/journal.pone.0036570)
Supplement: Table S3 — Additional statistics computed from individual bulk-PCR clone sequence datasets. EPD-PCR single infected individuals are greyed, others are super-infected. $ Category “Partly resolved” is not shown here but was always under 2.5% and can be deduced from the other values, “Partly resolved” = 100%-(“Unresolved”+”Resolved”). # Support is given as approximate likelihood ratio test (aLRT) values and is about the main bipartition observed in corresponding networks. NA: not assessed. (DOC) [file pone.0036570.s005.doc]

**Table S3. Additional statistics computed from individual bulk-PCR clone sequence datasets.** EPD-PCR single infected individuals are greyed, others are super-infected. ^$^ Category “Partly resolved” is not shown here but was always under 2.5% and can be deduced from the other values, “Partly resolved”=100%-(“Unresolved”+”Resolved”). ^#^ Support is given as approximate likelihood ratio test (aLRT) values and is about the main bipartition observed in corresponding networks. NA: not assessed.

|  | **Observed distance (%)** | | **Likelihood mapping^$^ (%)** | | **Phylogeny** |
| --- | --- | --- | --- | --- | --- |
| **Individual** | **Mean** | **Standard deviation** | **Unresolved *Star-like*** | **Resolved *Tree-like*** | **Support for main bipartition^#^** |
| B2 | 0.4 | 0.3 | 98.2 | 1.8 | NA |
| B3 | 0.6 | 0.4 | 96.2 | 3.8 | NA |
| B4 | 0.8 | 0.4 | 56 | 44 | NA |
| T3 | 1 | 0.7 | 72.3 | 27 | NA |
| B1 | 1.9 | 1.3 | 32.5 | 67.5 | 1 |
| T1 | 1.5 | 1.1 | 57.6 | 42.3 | 0.99 |
| T2 | 1 | 0.9 | 72.6 | 26.8 | 0.95 |
| T4 | 1.4 | 0.8 | 32.6 | 64.9 | 0.97 |
| T5 | 1.4 | 0.9 | 47.7 | 51.4 | 0.99 |
| T6 | 1.2 | 1.1 | 77.3 | 22.7 | 1 |
