# Supplementary material for: Detection of Retroviral Super-Infection from Non-Invasive Samples
Source: PLoS One. 2012 May 8;7(5):e36570. doi: 10.1371/journal.pone.0036570 (PMC3348140; doi:10.1371/journal.pone.0036570)
Supplement: Table S4 — Identification of EDP-PCR founder sequences from bulk-PCR clone sequence analyses where replication-based identification is not applicable. Only the most conservative view (that showing 100% accuracy in identification) is presented here. Outgroup probabilities (OPs) were computed with TCS for all possible combinations of bulk-PCR clone alignments which would not have allowed using replication as a criterion for EPD-PCR founder sequence identification. * marks a case where the founder sequences built separate networks consisting of less than 3 sequences; these were assumed to be negative. (DOC) [file pone.0036570.s006.doc]

**Table S4. Identification of EDP-PCR founder sequences from bulk-PCR clone sequence analyses where replication-based identification is not applicable.**

Only the most conservative view (that showing 100% accuracy in identification) is presented here. Outgroup probabilities (OPs) were computed with TCS for all possible combinations of bulk-PCR clone alignments which would not have allowed using replication as a criterion for EPD-PCR founder sequence identification. * marks a case where the founder sequences built separate networks consisting of less than 3 sequences; these were assumed to be negative.

|  |  |  | **Combination of three PCR products without replication of EPD-PCR founder sequence(s)** | | **Combination of four PCR products without replication of EPD-PCR founder sequence(s)** | |
| --- | --- | --- | --- | --- | --- | --- |
| **Individual** | **Infection status according to EPD-PCR analysis** | **EPD-PCR founder sequence later identified as replicated clones (# PCR products)** | **Number of possible combinations** | **Occurences of EPD-PCR founder sequences in the first, or first and second, OP** | **Number of possible combinations** | **Occurences of EPD-PCR founder sequences in the first, or first and second, OP** |
| **B1** | super-infection |  |  |  |  |  |
| **B2** | single infection | a (5) | - | - | - | - |
| **B3** | single infection | a (4) | - | - | - | - |
| **B4** | single infection | a (3) | 3 | 2 | - | - |
| **T1** | super-infection | b (2) | 6 | 1* | 2 | 0 |
| **T2** | super-infection | a (3) | 3 | 3 | - | - |
| **T3** | single infection | a (3) | 3 | 3 | - | - |
| **T4** | super-infection | b (2) | 6 | 3 | 2 | 1 |
| **T5** | super-infection | a (2) | 6 | 6 | 2 | 2 |
|  |  | b (2) | 6 | 4 | 2 | 1 |
| **T6** | super-infection | a (5) | - | - | - | - |
|  |  | **Summary** | 33 | 22 | 8 | 4 |
|  |  | **Success rate (%)** | 67 | | 50 | |
